# Research progress on the pharmacological effects of matrine

**DOI:** 10.3389/fnins.2022.977374

**Published:** 2022-08-23

**Authors:** Yanan Sun, Lu Xu, Qihan Cai, Mengmeng Wang, Xinliang Wang, Siming Wang, Zhiyu Ni

**Affiliations:** ^1^College of Traditional Chinese Medicine, Hebei University, Baoding, China; ^2^School of Basic Medical Science, Hebei University, Baoding, China; ^3^Affiliated Hospital of Hebei University, Baoding, China; ^4^Clinical Medical College, Hebei University, Baoding, China; ^5^Hebei Collaborative Innovation Center of Tumor Microecological Metabolism Regulation, Baoding, China

**Keywords:** matrine, pharmacological action, toxicology, pharmacokinetics, gut and gut microbes

## Abstract

Matrine possesses anti-cancer properties, as well as the prevention and treatment of allergic asthma, and protection against cerebral ischemia-reperfusion injury. Its mechanism of action may be (1) regulation of cancer cell invasion, migration, proliferation, and cell cycle to inhibit tumor growth; (2) reduction of oxidized low-density lipoprotein and advanced glycation end products from the source by exerting anti-inflammatory and antioxidant effects; (3) protection of brain damage and cortical neurons by regulating apoptosis; (4) restoration of the intestinal barrier and regulation of the intestinal microbiota. This article aims to explore matrine’s therapeutic potential by summarizing comprehensive information on matrine’s pharmacology, toxicity, and bioavailability.

## Introduction

**FIGURE 1:**
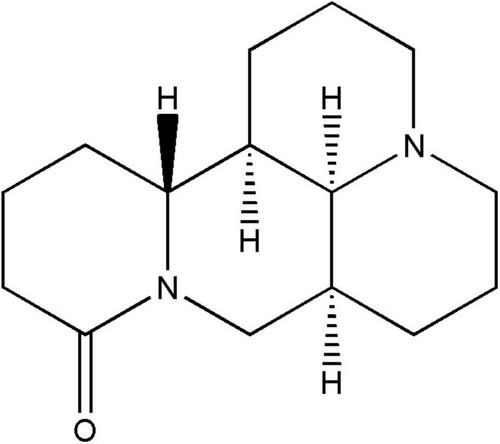
Chemical structure of matrine.

The dry roots of Leguminosae, Sophora herbs or subshrubs, *Sophora flavescens* Alt., and *Sophora tonkinensis*. They are widely used in clinical practice due to their ability to treat various damp-heat syndromes, carbuncle sores, and other heat-toxic syndromes and skin itching (Li et al., 2021). Matrine structure (MT) is shown in Figure 1, it is the main bioactive component of *Sophora flavescens* Alt. and *Sophora tonkinensis* roots (Gu et al., 2019; You et al., 2019), it can easily penetrate the biofilm barrier and produces a wide range of pharmacological effects (Table 1), including anti-cancer, anti-inflammatory, antioxidant, neuroprotective, etc., (You et al., 2020). However, the toxicity and bioavailability of MT in clinical usage remain unclear, and its absorption, distribution, metabolism, and excretion *in vivo* can affect its future development and application. Therefore, it is urgent to clarify the mechanism of MT as well as its toxicity and pharmacokinetics. This article explores matrine’s therapeutic potential by summarizing recent advances in the pharmacology, toxicology, and pharmacokinetics of MT, with an emphasis on its mechanism of action. Finally, the prospect of MT has been discussed.

**TABLE 1:** Pharmacology of matrine.

Pharmacological effect	Cell lines/Model	Activity/Mechanism(s) of action	Application	Reference
Anticancer effects	A549, H1975, and HCC827 cells	Suppresses the EGFR signaling pathway	*In vitro* and *in vivo*	Li et al., 2018
	MKN-45, BGC823, and SGC7901 cells	Targets EGFRp-Tyr845 and inhibits the EGFR-related signaling pathways	*In vitro*	Guo et al., 2015
	MCF-7 and MDA-MB-231 cells	Induces mitochondrial-mediated apoptotic pathway	*In vitro*	Wu et al., 2017b
	Hela and C33A cells	Downregulating the p38 signaling pathway	*In vitro*	Wu et al., 2017c
	A549 cells	Induces apoptosis and changes the miRNA expression profiles	*In vitro*	Liu et al., 2014
Anti-inflammatory effect	Ox-LDL-induced inflammatory injury of macrophages	Inhibits the MKKs/p38 MAPK signaling pathway	*In vitro*	Zhou et al., 2019
	Sensitization and stimulation of BALB/c mice with ovalbumin	Suppresses eotaxin and Th2 cytokine production	*In vivo*	Huang et al., 2014
	LPS-induced mastitis in mice	Affects NF-κB and MAPKs signaling pathways	*In vivo*	Yang et al., 2014
	LPS-induced BV2 microglia cells	Inhibits nuclear factor–kappa B and mitogen-activated protein kinase activation	*In vitro*	Dong et al., 2013
	Traumatic brain injury rat model	Inhibits Toll-like receptor 4/nuclear factor kappa-B-dependent inflammatory responses	*In vivo*	Dong et al., 2011
	LPS-activated BV2 microglial cells	Decreases the expression and release of HSP60	*In vitro*	Zhang et al., 2017
Antioxidant effect	I/R injury rat model	Activates the Nrf2/HO-1 pathway	*In vivo*	Jiang et al., 2015a
	As_2_O_3_-induced liver injury rat model	Activates the Nrf2/HO-1 signaling pathway	*In vivo*	Li et al., 2017
	High-fructose diet-induced steatohepatitis rat model	Enhances antioxidant and anti-inflammatory defense involving Nrf2 translocation	*In vivo*	Zhang et al., 2013a
	CCl_4_ -induced liver injury adult male mice model	Improves GSH, CAT, and GST level	*In vivo*	Khan et al., 2019
	Rat aortic endothelial cells	Restores the activity of p38MAPK/Nrf2/ARE	*In vitro* and *in vivo*	Liu et al., 2017b
Neuroprotective Effect	Focal cerebral ischemia mice and NMDA-induced neurotoxicity	Regulates NMDARs to overactivates NR2B	*In vitro* and *in vivo*	Zhang et al., 2013b
	Diabetic rats	Exerts anti-inflammatory and antioxidant effects	*In vivo*	Wang and Jia, 2014
	Diabetic mice	Inhibits endoplasmic reticulum stress and modulates the PK2/PKRs pathway	*In vivo*	Zhang et al., 2022
	Hypoxic-ischemic brain injury model in rats	Regulates p-Akt/GSK3β/HO-1/Nrf-2 signaling pathway	*In vivo*	Ge et al., 2018
	Thermal injury mice	Modulates JNK-mediated caspase-3 and BDNF/VEGF signaling	*In vivo*	Khan et al., 2020
	Alzheimer’s disease rat	Modulates the balance of Th17/Treg cytokines	*In vivo*	Zhang et al., 2015
	Scopolamine-induced amnesia model in mice	Inhibits AChE/BuChE and oxidative stress	*In vivo*	Sun et al., 2019a
	EAE model	Activates the PI3K/Akt/mTOR signaling pathway	*In vivo*	Liu et al., 2017a
	EAE model	Induces an A2 astrocyte phenotype	*In vivo*	Jing et al., 2021
	Chronic spinal cord injury model	Promotes neural circuit remodeling	*In vivo*	Tanabe et al., 2019
Effects on the gut and gut microbes	Diabetic mice model	As a CaSR agonist promotes intestinal GLP-1 secretion	*In vivo*	Guo et al., 2021
	CCl_4_-induced cirrhotic rats	Improves intestinal barrier function *via* NF-κB-mediated signaling pathway	*In vivo*	Wen et al., 2014
	DSS-induced murine colitis model	Improves gut barrier integrity, inhibits the PPAR-α signaling pathway, and modulates gut microbiota	*In vivo*	Yao et al., 2021
	LPS-stimulated mice	Enhances the expression of CCR7	*In vivo*	Wu et al., 2017a
	Healthy mice	Increases the number of beneficial bacteria	*In vivo*	Wu et al., 2021

## Pharmacological effects of matrine

### Anticancer effects

The treatment of cancer is a growing problem in much of the world and is becoming increasingly urgent. Cancer is characterized by uncontrolled tumor cell proliferation caused by the abnormal activity of various cyclins; therefore, cell cycle regulators are considered attractive targets in cancer therapy (Otto and Sicinski, 2017). MT can induce a G0/G1 cycle arrest in human non-small cell lung cancer cells by downregulating the activity of the epidermal growth factor receptor (EGFR)-Akt signaling pathway and inhibiting the growth of human non-small cell lung cancer cells; MT can inhibit tumor volume *in vivo* and weight, with inhibition rates of tumor transplanted mice were 16.29 and 35.35%, respectively (Li et al., 2018). MT inhibits gastric cancer cell proliferation in a dose-dependent manner by arresting cells in the G1 phase; it significantly inhibits gastric cancer cell migration and invasion by inhibiting EGFR phosphorylation (Guo et al., 2015). As can be shown, EGFR is an important anti-tumor target of MT. The viability and single-cell proliferation of breast cancer cells was significantly inhibited after MT treatment in a time- and concentration-dependent manner; additionally, the cell cycle of MT-treated breast cancer cells was arrested in the S phase and induced mitochondrial-mediated apoptosis, indicating that MT plays an important role in inhibiting breast cancer cell carcinogenesis (Wu et al., 2017b).

Matrix metalloproteinase(MMP)-2 and -9 are two related zinc-dependent endopeptidases that are critical in cancer cell invasion and migration, and MMP-2 and -9 are expressed at high levels in cancer tissues and invasive cell lines (Chang et al., 2015). The study found that intraperitoneal injection of MT could significantly inhibit tumor growth after transplanting cervical cancer cells into nude mice; it reduces the expression and activity of extracellular matrix factor, MMP-2, and MMP-9 by inhibiting the p38 signaling pathway, and inhibits the growth and metastasis of cervical cancer (Wu et al., 2017c). MT inhibits the proliferation of human non-small cell lung cancer cells, which induces apoptosis by altering miRNA expression profiles (Liu et al., 2014). In addition to the cancers listed above, it inhibits colorectal cancer (Ge et al., 2016), neuroblastoma (Mallepalli et al., 2019), etc.

### Anti-inflammatory effect

Oxidized LDL increases macrophage inflammatory response by activating reactive oxygen species (ROS)-mediated MKKs/p38 MAPK signaling pathway; MT can significantly reduce intracellular ROS production and further inhibit the activation of the MKKs/p38 MAPK signaling pathway to suppress oxidized low-density lipoprotein-induced inflammation (Zhou et al., 2019). *In vivo* experiments, BALB/c mice sensitized to ovalbumin-induced allergic asthma. Human tracheal epithelial cells were pretreated with MT and then cultured with TNF-α *in vitro*; it was found that MT can significantly reduce airway hyperresponsiveness in asthmatic mice, inhibit goblet cell hyperplasia, eosinophil infiltration, and inflammatory response in mice lung tissue; and can reduce the production of pro-inflammatory cytokines in human tracheal epithelial cells (Huang et al., 2014). Mouse mastitis was induced by lipopolysaccharide and MT was intraperitoneally injected before and after lipopolysaccharide induction, which could inhibit NF-κB signal path NF-κB p65 and I κ The phosphorylation of B decreased the phosphorylation of p38, ERK and JNK of mitogen activated protein kinases (MAPKs) signal pathway, and significantly reduced the damage of LPS to mammary gland (Yang et al., 2014). Similarly, MT treatment of BV2 microglia reduced inflammatory mediator levels following LPS stimulation by inhibiting NF-κB, ERK 1/2, p38MAPK, and JNK activation in activated microglia. This finding suggests that MT may provide a beneficial role in the treatment of inflammatory brain injury (Dong et al., 2013). Intraperitoneal injection of MT into traumatic brain injury rats found that MT can inhibit the Toll-like receptor 4/NF-κB-dependent inflammatory response, reducing neuronal apoptosis after traumatic brain injury (Dong et al., 2011). A similar finding was obtained by the treatment of MT in the brain neuroinflammation model established by injecting lipopolysaccharide into the hippocampus of rats, indicating that MT may effectively treat neuroinflammation in the brain (Mao et al., 2012). Extracellular release of heat shock protein 60 (HSP60) increases other pro-inflammatory effects by binding to Toll-like receptor 4 (TLR-4) and stimulating neuronal cell death generation factors (Zhang et al., 2012; Cheng et al., 2014). By inhibiting the HSP60 signaling pathway, MT can prevent microglia activation (Zhang et al., 2017).

### Antioxidant effect

Renal ischemia-reperfusion produces excess ROS, such as superoxide radicals, hydrogen peroxide, and hydroxyl radicals, and down-regulates the expression of some endogenous antioxidant enzymes, resulting in oxidative stress in the kidney, which MT can significantly reverse. Reperfusion-induced decrease in superoxide dismutase (SOD) activity and increase in malondialdehyde (MDA) levels suggest that MT can alleviate oxidative stress by enhancing endogenous antioxidant capacity and reducing lipid peroxidation (Jiang et al., 2015a). MT can inhibit As2O3-induced liver pathology and reduce liver ROS and MDA levels in a dose-dependent manner, which protects As2O3-induced oxidative damage *via* the Nrf2/HO-1 signaling pathway (Li et al., 2017). By promoting the translocation of Nrf2 to the nucleus and thereby up-regulating the expression of antioxidant enzymes and improving antioxidant activity, MT can effectively prevent the transformation of high-fructose diet-induced hepatic steatosis into non-alcoholic steatohepatitis in rats (Zhang et al., 2013a). MT can also increase glutathione (GSH), catalase (CAT), and glutathione mercaptotransferase (GST) levels in the prefrontal cortex and hippocampus of mice, while reducing MDA and nitrite levels, reducing the oxidative stress induced by CCl4 (Khan et al., 2019). Both *in vivo* and *in vitro*, advanced glycation end-products can lead to excessive production of ROS, induce apoptosis of vascular endothelial cells, and inhibit the antioxidant signal transduction of p38MAPK/Nrf2/ARE; it was found that the use of matrine alkaloids could restore the activity of p38MAPK/Nrf2/ARE, and p38MAPK kinase MKK3/6 can be considered as the molecular target of matrine alkaloids. Matrine alkaloids exert a protective effect on ROS-mediated apoptosis induced by advanced glycation end-products by targeting MKK3/6 and enhancing its phosphorylation (Liu et al., 2017b).

### Neuroprotective effect

The pharmacological mechanism of MT was studied by detecting the pharmacological properties of MT against focal cerebral ischemia *in vivo* and the neurotoxicity induced by N-methyl-D-aspartic acid (NMDA) *in vitro*. It was found that MT could regulate NMDA receptors (NMDARs) through the Bcl-2 and Bax families to over-activate NR2B and cause apoptosis, thereby protecting brain injury *in vivo* and cortical neurons *in vitro* (Zhang et al., 2013b). Some studies have reported that MT exhibits an analgesic effect on the vincristine-induced neuropathic pain mouse model that is mediated by regulating endogenous antioxidant defense mechanism and pro-inflammatory cytokines (Linglu et al., 2014; Gong et al., 2016). In addition, the anti-inflammatory and antioxidant effects of MT can significantly improve the learning and memory function of diabetic rats (Wang and Jia, 2014). Another study found that MT reduces cognitive impairment in diabetic mice by inhibiting endoplasmic reticulum stress and regulating the PK2/PKRs pathway (Zhang et al., 2022). By inhibiting apoptosis and oxidative stress, MT has a potential neuroprotective effect on cerebral ischemia-hypoxia injury in rats, and its mechanism may be related to Akt and GSK3β and the regulation of the Nrf-2/HO-1 signaling pathway (Ge et al., 2018). MT can also play a role in cerebral ischemia-reperfusion injury in rats, significantly reducing infarction and edema, and improving neural function after cerebral ischemia-reperfusion (Li et al., 2011). Similarly, MT pretreatment could significantly reduce infarct size and improve neurological function scores; it can also reduce the percentage of apoptotic neurons and morphological damage of neurons (Zhao et al., 2015). Burned mice exhibit anxiety- and depression-like behaviors, whereas MT exerts significant anxiolytic and antidepressant effects by inhibiting JNK-mediated apoptosis/inflammatory signaling, oxidative stress, and reversing burn-induced hippocampal BDNF/VEGF downregulation (Khan et al., 2020).

Matrine structure can restore Th17/Treg cytokine balance and alleviate cognitive impairment in Alzheimer’s disease rats in a dose-dependent manner (Zhang et al., 2015); MT can improve scopolamine-induced amnesia by inhibiting acetylcholinesterase (AChE) and butyrylcholinesterase (BuChE) activities (Sun et al., 2019a). Meng et al. (2017) demonstrated that MT has a neuroprotective effect against MPTP-induced Parkinson’s disease. MT has an analgesic effect on neuropathic pain caused by chronic contractile injury; however, its mechanism requires more molecular and cellular research (Haiyan et al., 2013). MT can effectively suppress the severity of experimental autoimmune encephalomyelitis (EAE), increase myelin protein production in the central nervous system, and activate the corpus callosum P13K/Akt/mTOR signaling pathway (Liu et al., 2017a). In addition, MT can protect stressed oligodendrocytes by inhibiting apoptosis and enhancing mitochondrial autophagy, so achieving the purpose of effective treatment of EAE (Wang et al., 2019). In the CNS of EAE, MT inhibits A1 production and promotes neuroprotective A2 production; MT also downregulates the expression of vascular endothelial growth factor-A and upregulates the expression of the tight junction proteins Claudin 5 and Occludin, thereby protecting the BBB from damage caused by CNS inflammation (Jing et al., 2021).

In addition to the above findings, MT can increase synaptic density and promote the remodeling and reconnection of neural circuits (Tanabe et al., 2019). Kan et al. (2017) discovered that MT significantly induced the expression of camp and PKA in cells and promoted axonal regeneration; and cAMP blockade significantly reduced MT-induced PKA expression and production of BDNF, which is an effective neurotrophic factor for nerve regeneration, indicating that MT can directly promote regeneration of the injured central nervous system.

### Effects on the gut and gut microbes

Glucagon-like peptide-1 (GLP-1) released from intestinal endocrine cells, controls dietary-related blood glucose fluctuations by increasing insulin and inhibiting glucagon secretion. GLP-1 also inhibits gastric emptying and food intake, maximizes nutrient absorption, and limits weight gain (Drucker, 2018). MT can act as a calcium-sensing receptor (CaSR) agonist and stimulate GLP-1 intestinal secretion (Guo et al., 2021). Through NF-κB-mediated signaling, MT can reverse CCl4-induced histological changes and restore intestinal barrier integrity (Wen et al., 2014). MT was also found to improve gut barrier integrity in mice with dextran sulfate-induced ulcerative colitis; it also altered gut microbiota composition and function, such as increased human *Pasteurella enterica* abundance and reduced *Helicobacter gammanii* abundance, to achieve the purpose of improving colitis (Yao et al., 2021). LPS-induced intestinal inflammation and oxidative balance were improved by MT, as was the expression of the intestinal chemokine receptor 7 (CCR7), and CCR7 siRNA transfection inhibited MT’s protective effect (Wu et al., 2017a).

Biofilm formation is linked to bacteria’s perception of their surroundings and bacterial density (Kai, 2018), and quorum-sensing systems are lacking in bacterial biofilms (Kumar et al., 2016). MT has a biofilm inhibitory effect, diminishing E. coli resistance in terms of inter-community communication, and drug-resistant strains treated with MT showed reduced adhesion, reducing *E. coli* pathogenicity (Sun et al., 2019b). Different administration routes of MT can cause differences in the intestinal flora. MT delivered intraperitoneally is more favorable to improving the structure and function of intestinal flora than MT administered *via* gavage. Intraperitoneal MT injection can significantly change the structure of Kunming mice’s intestinal flora, increase the colonization of beneficial bacteria Lactobacillus acidophilus in the intestinal tract, and cause differences in metabolic pathways such as glycan biosynthesis and metabolism, transport, and catabolism. The identification of the mechanism by which MT exerts its pharmacodynamic effect laid the foundation. MT does not affect the structure of the intestinal flora in mice, unlike the steady decline in the diversity and richness of the intestinal flora in mice induced by the overuse of antibiotics such as amoxicillin. However, amoxicillin changes the composition and structure of the microbial community by increasing the ratio of pathogenic bacteria to beneficial bacteria, whereas MT increases the number of beneficial bacteria, and provides a rationale for developing antibiotic alternatives to reduce bacterial resistance and gut flora imbalance (Wu et al., 2021).

In other animal models, such as mice, trinitrobenzene sulfonic acid can induce intestinal inflammation, colon damage, and intestinal microbiota dysbiosis, and MT significantly improves the intestinal microbiota community and protects intestinal tissue (Li et al., 2019).

## Toxicity of matrine

Matrine structure has a wide spectrum of pharmacological effects; however, its toxic effects limit its clinical application (Table 2).

**TABLE 2:** Toxicity of matrine.

Cell lines/Model	Dose	Activity/Mechanism(s) of action	Application	Reference
Human hepatocytes	7, 14, and 21 mg/L, 72 h	Enhances ECOD activity and reduces cytokine-induced NO_2_^–^ levels	*In vitro*	Gong et al., 2015
	140 mg/L, 72 h	Induces the expression of CYP2A6, CYP2B6, and CYP3A4 proteins		
	250, 500, and 1,000 mg/L, 72 h	Reduces the levels of LDH and AST		
BALB/c mice	50 and 100 mg/kg/day for 7 days	Induces ROS production and inhibits mitochondrial membrane potential and ATP level	*In vivo*	Liu et al., 2020
LO2 cells	6, 12, and 18 mmol/L, 24 h	Promotes the phosphorylation of JNK	*In vitro*	Gu et al., 2018
HL-7702 cells	0–4 mg/mL, 24 h	Inhibits Nrf2 pathway, activates ROS-mediated mitochondrial apoptosis pathway and arrests cell cycle at the S phase	*In vitro*	You et al., 2019

Aside from dosage, it is unreasonable to discuss the toxicity of any drug, and MT is no exception. A low concentration of matrine (7, 14, and 21 mg/L) enhances ethoxycoumarin-O-demethylation (ECOD) activity and reduces cytokine-induced NO2- levels in human hepatocytes. For 72 h, a moderate dosage of matrine (140 mg/L) induced the expression of CYP2A6, CYP2B6, and CYP3A4 proteins, indicating that it can regulate the CYP450 enzyme system to protect hepatocytes. In addition, high concentrations of matrine (250, 500, and 1,000 mg/L) reduced the levels of lactate dehydrogenase (LDH) and aspartate aminotransferase (AST), and cytotoxicity to hepatocytes, resulting in decreased cell viability and total protein content (Gong et al., 2015). Increased taurine in urine is a biomarker for liver injury caused by several drugs (Wang et al., 2013; Li et al., 2014). Thus, elevated taurine levels in rat urine suggest that high-dose MT induces liver dysfunction and damage (Li et al., 2015). At hepatotoxic doses, MT caused centrilobular hypertrophy of the mouse liver; in addition, other matrine alkaloids were more toxic in combination with MT than MT alone (Gu et al., 2019). Caspase-3 and caspase-9 activation was observed in normal mouse hepatocytes treated with MT; after intraperitoneal injection of MT into healthy mice, vacuolar degeneration was found in the cytoplasm of mouse liver tissue. Furthermore, high concentrations of matrine (500 and 1,000 mg/L) were shown to induce ROS production and inhibit mitochondrial membrane potential and ATP level, which may be related to matrine toxicity to hepatocytes (Liu et al., 2020). It can be seen that changing the concentration of matrine has an opposite effect on antioxidation. The effects of endoplasmic reticulum stress and ROS-mediated JNK phosphorylation are linked to MT-induced cellular injury in human normal hepatocytes. Antioxidants, JNK phosphorylation inhibitors, and endoplasmic reticulum stress inhibitors may be potential antidotes to MT and Chinese medicine containing MT-induced liver damage (Gu et al., 2018). Past studies have demonstrated that the hepatotoxic effect of MT is exerted by inhibiting the Nrf2 pathway, activating the ROS-mediated mitochondrial apoptosis pathway, and arresting the cell cycle in the S phase (You et al., 2019). However, the antioxidant N-acetylcysteine reverses MT-induced hepatotoxicity and ROS production (Liu et al., 2020).

In addition to being hepatotoxic, MT has also been shown to be developmental and neurotoxic to zebrafish embryos, as well as teratogenic and lethal (Lu et al., 2014). MT depresses the central nervous system and impairs balance and coordination in ICR mice when administered at doses of 10 and 40 mg/kg/day for 60 days.

## Pharmacokinetics of matrine

In addition to being hepatotoxic, the bioavailability of MT is not ideal and has a short *in vivo* half-life (Jiang et al., 2015b). The pharmacokinetics of MT in rats were studied using the UPLC-MS/MS method, and it was found that at the dose of 2 mg/kg, its absolute oral bioavailability was 17.1 ± 5.4% (Yang et al., 2010). Human organic cation transporters (hOCTs; SLC22) are highly expressed in the intestine, liver, kidney, heart, brain, and other organs, and are primarily responsible for the absorption, distribution, and elimination of endogenous and exogenous substances. When the inhibitory effect of MT on the functions of hOCT1 (SLC22A1), hOCT2 (SLC22A2), and hOCT3 (SLC22A3) at 100-fold excess, it was found that MT did not significantly inhibit hOCT1, hOCT2, and hOCT3. At 6 mM, MT, on the other hand, exhibited an 88% inhibitory effect on hOCT3-mediated substrate uptake. These data suggest that drug interactions may occur during hOCT3-mediated intestinal absorption (Pan et al., 2014). The protein binding rate of MT in plasma is very low (5.10–10.55%), causing the drug to metabolize quickly and be eliminated from the body (Tang et al., 2013). AUC, Cmax, and Tmax are the key parameters of pharmacokinetics, the former reflects the degree of drug absorption, and the latter two comprehensively reflect the absorption, distribution, metabolism, and excretion of drugs. The pharmacokinetics of MT in organs and tissues such as liver, blood, and skin were studied after intravenous injection (40 mg/kg) or transdermal administration (6 mg/cm^2^, 5 cm^2^) in rats. AUC (0-t) values in the liver, Micro dialysate, and plasma after intravenous administration were 395.91 ± 74.48, 848.86 ± 146.35, and 1304.07 ± 305.92 min mg/l respectively; after transdermal administration (Tang et al., 2017). The difference in AUC indicates that the blood concentration of MT will be affected by different administration methods. These data allow us to better understand the transdermal pharmacokinetics of MT, which will contribute to further clinical and laboratory studies.

## Conclusion and future prospects

Matrine structure can regulate the secretion of a variety of inflammatory factors and regulate the activation of multiple signaling pathways, indicating that MT’s mechanism of action is complex and has multiple targets. This article reviews the pharmacological effects and toxicities of MT and provides a reference for the treatment of related diseases. The main function of the matrine is to affect the intestinal tract and intestinal microorganisms. Matrine, as a calcium-sensitive receptor agonist, can activate intestinal tissue and restore the integrity of the intestinal barrier *via* the NF-κB-mediated signal pathway. In addition, it will also affect the composition and function of intestinal microbiota.

The brain-gut axis is a regulatory system for two-way signal communication between the nervous system and the gastrointestinal tract, and the digestive tract’s microbial community is an important participant in the brain-gut axis. Changes in intestinal information will be transmitted to the central nervous system through the vagus nerve, sympathetic nerve, etc. Matrine, as a result, has a therapeutic effect on central nervous system diseases.

To prevent inflammation, the intestinal flora stimulates the afferent neurons of the intestinal nervous system. Therefore, Matrine’s effect on intestinal flora contributes to its anti-inflammatory effect.

Increased proinflammatory cytokines production causes increased oxidative and nitrosative brain damage. Matrine’s anti-inflammatory effect also enables it to play as an antioxidant.

**FIGURE 2:**
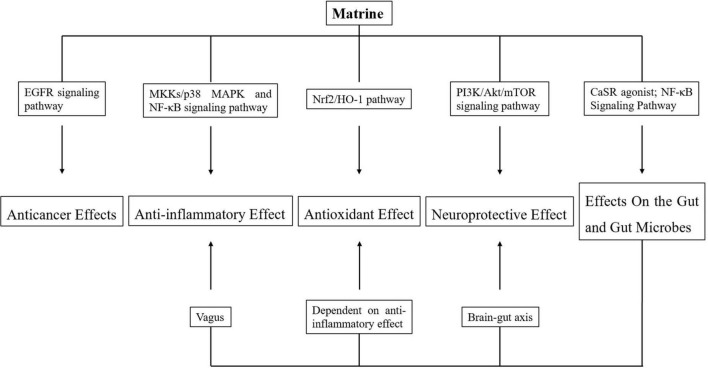
Pharmacological activities of matrine.

Finally, the pharmacological effect of matrine cannot be exerted without considering its impact on the intestine and intestinal microorganisms (Figure 2).

These pharmacological effects suggest that MT may have potential application value in psychiatric diseases such as depression. However, from the standpoint of the brain-gut axis, the current treatment of MT for depression has not been elucidated. Therefore, further exploring whether MT can improve depression through the brain-gut axis may have important significance for the clinical application of MT and the intervention of depression.

## Author contributions

YS contributed to the overall organization of this review and the preparation of figures and tables. YS, LX, QC, MW, and XW contributed to writing. All authors contributed to the article and approved the submitted version.
